# Evaluation of Faecal Microbiota Following Probiotics in Infants of Mothers with Gestational Diabetes Mellitus Trial: Protocol for Double-Blind Placebo-Controlled Randomized Trial

**DOI:** 10.3390/microorganisms13010112

**Published:** 2025-01-08

**Authors:** Gayatri Athalye-Jape, Chandra Prakash Rath, Harshad Panchal, Archita Mishra, Dorothy Graham, Sanjay Patole

**Affiliations:** 1 Department of Neonatology, King Edward Memorial Hospital, Perth, WA 6008, Australia; chandra.rath@health.wa.gov.au (C.P.R.); harshad.panchal@health.wa.gov.au (H.P.); 2 School of Medicine, The University of Western Australia, Crawley, WA 6009, Australia; 3 The Kids Research Institute, Perth, WA 6009, Australia; archita.mishra@sydney.edu.au; 4 Indian Institute of Sciences, University of Sydney, Sydney, NSW 2050, Australia; 5 Department of Obstetric Medicine, King Edward Memorial Hospital, Perth, WA 6008, Australia; dorothy.graham@health.wa.gov.au

**Keywords:** probiotics, infants of diabetic mothers, gestational diabetes, microbiome

## Abstract

(1) Background: The incidence of gestational diabetes mellitus (GDM) is rising globally. The current evidence indicates that GDM, especially in conjunction with maternal overweight, can alter the composition of infants’ gut microbiota, potentially increasing the risk of inflammatory diseases, metabolic disorders, and neurodevelopmental issues later in life. Probiotic supplantation early in life might establish eubiosis and mitigate future complications. To best of our knowledge, no study has evaluated the effects of probiotics on gut dysbiosis in the infants of mothers with GDM. (2) Methods: This study will be a single-centre, double-blind, randomized, placebo-controlled trial enrolling sixty neonates born after 35 weeks of gestation to mothers with GDM. The participants will be randomly assigned to receive either a triple-strain probiotic or a placebo for four months. The primary objective is to assess the effectiveness of probiotic supplementation in correcting gut dysbiosis in the infants of mothers with GDM at four months of age. Faecal microbiome composition shall be estimated using 16SrRNA and shotgun sequencing. The secondary outcomes will include the quantification of faecal short-chain fatty acids at birth and at four months, as well as growth and developmental assessments at four, twelve, and twenty-four months. (3) Trial registration: This trial protocol is registered (ACTRN12624000930583p) in the Australian Clinical Trials registry (ANZCTR).

## 1. Introduction

With a rising trend in obesity worldwide, the incidence of gestational diabetes mellitus (GDM) and associated pregnancy and perinatal complications have increased [1]. The pooled global standardized prevalence of GDM, based on the International Association of Diabetes in Pregnancy Study Group criteria, is 14%, with a range from 0.7% to 36.8%. This prevalence shows little variation between high- and low-income countries [2]. Following the global trend, the incidence of GDM in Australia has increased from 5.2% in 2010 to 16.1% in 2018. This increase is attributed to factors such as advancing maternal age and a growing prevalence of overweight and obesity, with 27% of women classified as overweight and 22% as obese in 2020 [3].

Intestinal microflora and their metabolites are believed to play a crucial role in body weight regulation, energy homeostasis, fermentation, and the absorption of non-digestible carbohydrates. Mothers with GDM are known to have distinct microbial profiles in their gut, vagina, oral cavity, and placenta compared to those without GDM [4]. Dysbiosis may contribute to the development of metabolic disorders, including obesity, diabetes, and GDM [5]. The mother serves as the primary source of the infant’s microbiome, with the most substantial exposure occurring during birth and the post-partum period through both vertical and horizontal transmission [6,7]. Several studies suggest that GDM alone, or together with maternal overweight can alter the composition of infants’ microbiota and may set the stage for future risk of inflammatory and metabolic diseases [8,9]. The recent evidence suggests significant dysbiosis in infants born to mothers with GDM with reduced alpha diversity and an abundance of Firmicutes and Proteobacteria [10]. The disruption of microbiota early in life is known to be associated with the later onset of inflammatory, immune-mediated, allergic, and dysmetabolic diseases [11,12]. The alterations in neonatal microbiota associated with GDM may provide valuable insights into the increase in the intergenerational prevalence of obesity and diabetes. In addition, infants of GDM mothers are also at increased risk of atypical neurodevelopment, such as autism spectrum disorder [13]. A notable decrease in Prevotella genera abundance is observed in both children with autism with gastrointestinal symptoms, as well as infants born to mothers with GDM [14,15]. This could indicate a potential physiological explanation for the higher occurrence of autism in infants born to mothers with GDM. Hence, early establishment of a eubiotic environment is imperative. We have two approaches to address early life dysbiosis: administering probiotics to either the mother, or directly to the infant. The perinatal probiotic supplementation of pregnant women regardless of GDM status may be associated with an increased risk of pre-eclampsia [16,17]. To our knowledge, no studies have examined probiotic supplementation in infants of mothers with GDM to improve their gut microbial composition or clinical outcomes. Therefore, we propose a randomized controlled trial (RCT) to evaluate the effectiveness of probiotics in influencing the gut microbial composition of infants born to mothers with GDM.

## 2. Study Materials and Methods

### 2.1. Hypothesis and Aim

We aim to assess the effects of probiotic supplementation on the faecal microbiome composition of infants of mothers with GDM. We hypothesize that probiotic supplementation with triple-strain Bifidobacteria, 1 × 10^9^ CFU of each (*Bifidobacterium breve* M-16V, *Bifidobacterium longum* subsp. *infantis* M-63, *Bifidobacterium longum* subsp. *longum* BB536), will reduce dysbiosis compared to that of the control group. This will be assessed as an increase in the abundance of beneficial bacteria (e.g., bifidobacteria) and a reduction in the abundance of pathogenic bacteria (e.g., gammaproteobacteria).

### 2.2. Rationale for Selecting the Three-Strain Bifidobacteria Product

This approach is grounded in previous studies, highlighting the benefits of multi-strain probiotics and the importance of bifidobacteria in infants [18], and our two previously published RCTs using triple-strain Bifidobacteria [19,20].

One of these trials evaluated this product against a placebo in term neonates with congenital gastrointestinal surgical conditions [20]. The median (IQR) relative abundance of potentially pathogenic families was lower, and the relative abundance of Bifidobacteriacae was higher in the probiotic versus placebo groups after two weeks of supplementation [20]. Our other RCT compared a single- vs. three-strain bifidobacterial probiotic in extremely preterm infants [19]. The time to full enteral feed (primary outcome) was similar in infants administered single vs. three-strain bifidobacteria at a dose of 3 billion CFU/day. Both the probiotics were effective in reducing dysbiosis significantly [19]. Importantly, the probiotic product used in our trials was safe, and none of the participants developed sepsis due to the administered probiotic bacteria.

### 2.3. Participants

Infants of mothers with GDM born at >35 weeks and admitted to a postnatal ward.

### 2.4. Design and Setting

**Figure 1:**
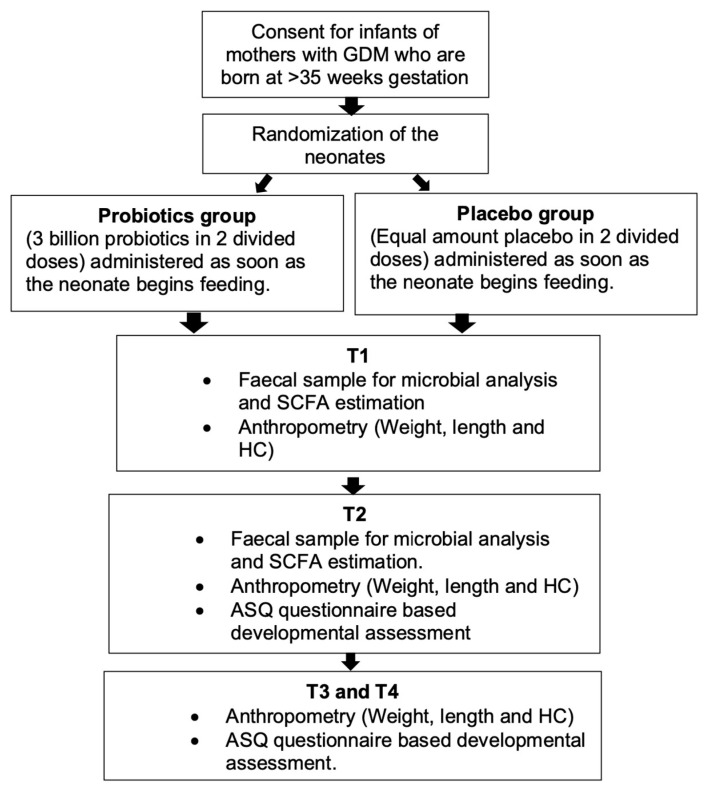
Study design (GDM: gestational diabetes mellitus; SCFA: short-chain fatty acid; ASQ: Ages and Stages Questionnaire; HC: head circumference; T1: first week of life; T2: after 4 months of probiotics supplementation; T3: at 12 months of age; T4: at 24 months of age).

Double-blind, randomized placebo-controlled trial in postnatal wards of our tertiary referral and teaching hospital in Western Australia. Refer to Figure 1 for design.

### 2.5. Eligibility Criteria

Infants will be eligible for recruitment if: gestation > 35 weeks, born to mothers with GDM, predominantly breast-fed (>80% feed as breast feed), maternal intention of breast feeding for at least 6 months, presence of informed parental consent, and resident of local metropolitan area.

### 2.6. Exclusion Criteria

Infants will be excluded if: presence of congenital malformation, chromosomal aberration, or immunodeficiency; residing outside the local metropolitan area; need for antibiotics at birth; presence of maternal chorioamnionitis or prolonged rupture of membranes (>18 h); or mothers with GDM administered antibiotics within 7 days prior to delivery.

### 2.7. Intervention

Triple-strain Bifidobacteria: 1 × 10^9^ CFU of each (*Bifidobacterium breve* M-16V, *Bifidobacterium longum* subsp. *infantis* M-63, *Bifidobacterium longum* subsp. *longum* BB536) administered from birth till 4 months of age.

### 2.8. Control

The infants will receive identical placebo sachets containing maltodextrin that match the weight and appearance of the probiotic sachet.

### 2.9. Sample Size Calculation

A sample size of 30 per group (total 60) was considered adequate to detect a 1.5-fold difference in alpha diversity with 80% power and a significance level of 0.05. By focusing on biological relevance, this sample size ensures that even subtle changes in microbial composition can be detected, providing a robust foundation for understanding the underlying biological mechanisms. This sample size allows for the detection of biologically meaningful variations in microbial composition, ensuring that differences in diversity metrics, such as Shannon diversity or Bray–Curtis dissimilarity, are statistically significant. By focusing on these well-defined biological parameters, this study can reliably capture important variations in microbial communities, providing a strong foundation for understanding their role in the system under investigation [21,22,23].

### 2.10. Primary Outcome

Faecal microbiome composition will be estimated using 16S rRNA and shotgun sequencing. Baseline gut dysbiosis will be assessed at Timepoint 1 (T1: first week of life) by evaluating the ratio of beneficial to pathogenic bacteria, with reductions in dysbiosis measured at Timepoint 2 (T2: after four months of supplementation) in the probiotics group compared to the placebo group. The following variables will be analysed: (1) the presence or absence of specific species or pathways and clusters based on the unsupervised clustering of the gut microbial profile; (2) alpha diversity, richness, the abundance of particular species or pathways, and the differences in these numeric variables (treatment vs. baseline).

### 2.11. Secondary Outcomes

Faecal short-chain fatty acids (SCFAs) and bile acids will be quantified in the first week of life (T1), and again four months after probiotic supplementation (T2). Growth parameters, including weight, length, and head circumference, will be assessed at 4 (T2), 12 (T3), and 24 (T4) months of age. Developmental parameters will be evaluated using the Ages and Stages Questionnaire (ASQ) and the ASQ-Social Emotional scale, 2nd version, at 4, 12, and 24 months of age.

### 2.12. Rationale for Continuing Probiotics for Four Months

The introduction of solid foods around four months of age is an important dietary event during infancy that causes profound shifts in the gut microbial composition towards a more adult-like state [24]. Hence, we decided to target the dysbiosis of infants of diabetic mothers, while they are still on an exclusively milk-based diet.

### 2.13. Rationale for Assessing Growth and Developmental Parameters

There is conflicting evidence in relation to the effects of probiotics on growth. A recent systematic review by our group showed that probiotic supplementation was associated with better short-term weight gain [25]. Probiotic supplementation had no effect on neurodevelopmental impairment. They recommended more adequately powered RCTs that focus on the growth and neurodevelopment of infants receiving probiotic [25]. Another systematic review comprising of 79 RCTs concluded that in otherwise healthy children aged 0–59 months, probiotics may have a small, but heterogenous effect on weight and height in low- and middle-income countries, but not in children from high-income countries [26]. In contrast, a different systematic review, where the mean duration of probiotic supplementation was 5.6 ± 2.84 months, found no significant effect on weight or height [27].

The gut microbiota–brain (GMB) axis influences brain development and function via immune signalling, the hypothalamo–pituitary axis (HPA), the vagus nerve, and neurotransmitters produced by gut bacteria and metabolites, such as SCFAs, tryptophan derivatives, and bile acids. A systematic review which included nine RCTs (n = 3026 participants) demonstrated no significant difference in neurocognitive outcomes in between those treated with probiotics or a placebo, except in a subgroup of patients who received probiotics for >6 months (2 RCTs, n = 451 participants) [28]. However, a specific microbial pattern has been shown to be associated with variable developmental outcomes. A recent report showed negative associations of gross motor development with the relative abundance of Klebsiella and improved gross and fine motor scores with Lactobacillus and Streptococcus abundance [29]. Similarly, another study reported that Turicibacter and Parabacteriodes were highly abundant in the below-median fine motor score group, while Collinsella, Caprococcus, Enterococcus, Fusobacterium, Holdemanella, Propionobacterium, Roseburia, Veilonella, Bifidobacteria, and Lactobacillus were more abundant in the above-median fine motor score group [30].

### 2.14. Safety Monitoring

This will be assessed by monitoring for abdominal distension, vomiting, and diarrhea, leading to the cessation of supplementation. The risk of probiotic sepsis is rare, as reported in a recent systematic review [31]. To the best of our knowledge, probiotic sepsis has not been reported in healthy full-term infants in the literature. Moreover, probiotic sepsis (with Bifidobacteria) is easy to detect with blood culture and treat with routine antibiotics like penicillin and vancomycin. Fatality due to probiotics in preterm infants has not been reported so far, except for two cases, including one where death was attributed to a contaminated probiotic product [31]. In the second case, it was unclear whether death was ‘associated with’ or ‘due to’ probiotic sepsis (https://www.fda.gov/safety/medical-product-safety-information/risk-invasive-disease-preterm-infants-given-probiotics-formulated-contain-live-bacteria-or-yeast; accessed on 23 September 2024).

The risk of probiotic sepsis and associated morbidity is minimised by the independent microbiological assessment of the probiotic under investigation, the ability to detect bifidobacteria in blood culture using our routine blood culture assessment, and the susceptibility of probiotic bacteria to prescribed antibiotics for late-onset sepsis.

Bi-weekly phone calls will be made by the research team to parents to enquire about any gastrointestinal adverse effects, such as the number of stools per day, difficulty in passing stool as the number of stools difficult to pass per day, and stool consistency recorded using a validated 5-point scale (1 = watery, 2 = runny, 3 = mushy soft, 4 = formed, and 5 = hard) provided in a pictorial representation to parents. The frequency of spitting-up/vomiting and flatulence episodes per day will be recorded on a categorical scale (1 time; 2–3 times; 4–6 times; >7 times). Categorical scales will also be used to assess the daily durations of crying or fussing (<10 min; 10–30 min; >30 min to 1 h; >1–2 h; >2–3 h; >3 h), sleeping (0–7, 8–12, 12–16, 16–20, 20–24 h), or the severity of spitting-up/vomiting (1 teaspoon or less; 1 tablespoon; 2 tablespoons; about half of the feeding; more than half of the feeding). Episodes of abdominal distension (present or absent) shall be recorded in a parent diary. All adverse events will be recorded and evaluated by the investigators for causality and severity. Adverse events will be considered as serious if they are life-threatening, cause permanent harm, or results in hospitalization. All other adverse events will be considered non-serious.

### 2.15. Establishment of a Data and Safety Monitoring Committee (DSMC)

A Data and Safety Monitoring Committee (DSMC) will be established. The committee will have the required expertise in paediatrics, neonatology, and trial design, but will not be involved in the care of study participants and would have no competing interests. The committee will monitor all the outcomes from recruitment until four months of age. The DSMC will review and approve the study protocol and will review all serious adverse events as they occur. No interim analyses are planned, but the DSMC will reserve the right to conduct an interim analysis or advise suspension or the termination of ongoing enrolment to this study.

### 2.16. Randomization, Allocation Concealment and Blinding

Group assignment will be allocated by a computer-generated, randomization sequence in randomly ordered block sizes of 2 and 4. Opaque, sealed, coded envelopes will be used for randomization. The neonates of multiple pregnancies will be considered as separate participants. Allocation concealment will be optimized by prescribing allocation only after informed parental consent and recording baseline neonatal data. The clinical trial pharmacist will supply the randomization sequence and sachets (identical design, weight, smell, and taste) containing either the triple-strain Bifidobacteria or placebo (equal volume of dextrin) manufactured by Morinaga Milk Industry Co., Ltd., Tokyo, Japan, to the healthcare personnel looking after the infants on the postnatal wards, and then to the parents. The parents will be educated about reconstituting the sachets and administering the probiotic or placebo at home and to monitor for any adverse effects. The hospital pharmacy will supply additional sachets to parents of participating infants as needed till the completion of the protocol (4 months of age). This will assure the masking of all investigators, clinical and non-clinical outcome assessors, midwifery and nursing staff, and the parents with regarding to the allocation of the enrolled infants.

### 2.17. Probiotic Protocol

Enrolled neonates will be supplemented with the freshly reconstituted contents of the allocated sachets every day, which will be continued till four months of age. The dry, lyophilized powder in the sachets will be reconstituted using the mums’ own milk (first choice) or sterile water for injection. The dose will be 3 × 10^9^ CFU/day (1 mL reconstituted solution twice daily). Supplementation will be temporarily withheld if vomiting, diarrhea, or abdominal distension is noted.

The manufacturer, Morinaga Milk Industry Co., Ltd., are not a sponsor and will only supply the probiotic and placebo free for the trial and are not involved in the design, conduct, analysis, and reporting of the trial.

### 2.18. Ethics Statement

Approval was obtained from the Child and Adolescent Health Service Ethics Committee at Perth Children’s Hospital prior to commencing recruitment (RGS0000007074) in October 2024.

### 2.19. Trial Registration

This trial protocol has been registered (ACTRN12624000930583p) in the Australian Clinical Trials registry (ANZCTR).

### 2.20. Data Handling, Storage, Confidentiality

The National Health and Medical Research Council (NHMRC) Australian guidelines will be followed for confidentiality and data storage [32].

### 2.21. Reporting

The revised CONSORT guidelines will be used for reporting the results as highlighted in the EQUATOR (Enhancing the QUAlity and Transparency Of health Research) network [33].

## 3. Methods of Sample Collection, Processing and Analyses

The methodology for the Probiotics in Infants of Mothers with gestational Diabetes mellitus (PRIMD) trial will be identical to that reported in our previous RCTs [19,20].

### 3.1. Faecal Sample Collection

Stool samples will be collected from the diaper at two time points in sterile containers from each infant. The first sample (T1) will be collected as soon as possible after birth/admission to the postnatal ward (week 1). The second sample (T2) will be collected at the age of 4 months. All the samples will be labelled, weighed, and stored at −80 °C. After completing recruitment, the samples will be shipped on dry ice to the laboratory for storage at −80 °C and analyses. Acidified samples (5-fold in 1% phosphoric acid) will be shipped on dry ice to the laboratory for SCFA analysis.

### 3.2. DNA Extraction

DNA will be extracted from the stool samples (0.3 g) using a Qiagen DNeasy PowerSoil Pro kit (Qiagen, Hilden, Germany, cat# 47014). However, instead of vortexing, the samples will be subjected to physical lysis in a bead-beater (FastPrep 24, MP Biomedicals, Irvine, CA, USA) for 1 min at 6.5 m.s. DNA will be eluted in molecular-grade water and stored at −80 °C.

### 3.3. Quantification of SCFA

A modified gas chromatography–mass spectrometry (GC-MS)-based method will be used to extract and analyse SCFAs from the faecal samples. Acetic acid, propionic acid, isobutyric acid, butyric acid, isovaleric acid, 4-methyl valeric acid (internal standard, IS), ethyl acetate, and meta-phosphoric acid will be purchased from Merck (Singapore). For sample preparation, 0.2 g of the faecal sample will be first diluted 5-fold in 1 mL of 1% phosphoric acid and frozen at −20 °C immediately after collection. Before SCFA extraction, the frozen samples will be thawed and added to 100 μL of 10% meta-phosphoric acid solution to adjust the pH to around 2. The samples will be homogenized by vortexing for about 10 min and centrifuged for 30 min at 20,817× *g*. After that, 1 mL of aqueous supernatant will be transferred into a new tube, and 4-methyl valeric acid will be added to achieve a final concentration of 500 μM. Then, the mixture will be vortexed for 30 min and centrifuged for 10 min at 20,817× *g* after addition of 500 μL of ethyl acetate. Then, 2 μL of the organic extracts will be injected in splitless mode into an Agilent GC-MS system (Agilent Technologies (Santa Clara, CA, USA) 7890B-5977B Bundle with Stainless Steel Source) and separated using an HP-FFAP capillary column (30 m × 0.250 mm × 0.25 μm; Agilent, Santa Clara, CA, USA). Helium will be used as the carrier gas at 1 mL/min. The column temperature will initially be 80 °C (1 min), and then increased to 120 °C at 20 °C/min, and finally to 210 °C at 6.13 °C/min and kept at this temperature for 2 min. The solvent delay will be 3.5 min. The detector will be operated in SIM acquisition mode with 30–250 *m/z* range. The injector, ion source, quadrupole, and interface will be set at 250 °C, 230 °C, 150 °C, and 280 °C, respectively. SCFAs will be identified by comparing with the standards and double-confirmed with the NIST 17 library. Quantification will be performed using Mass Hunter Quantitative software (version B.09.00), with base peak ion selected as the quantifier for each SCFA. Calibration graphs will be constructed by plotting relative response (ratio of peak area of SCFA/peak area of IS) versus relative concentration for each SCFA. Final concentrations will be expressed as microgram of SCFA per gram wet weight faecal sample. All analyses will be performed in duplicate. Wilcoxon rank analysis will be performed to compare the SCFA concentrations between the different groups.

### 3.4. Linearity and Sensitivity of SCFA

A stock solution containing a mixture of standards (20 mM final concentration each) in ethyl acetate will be diluted to obtain a calibration curve ranging from 2 to 15,000 μM. This will be added to each diluted standards mixture (500 μM final concentration). Calibration graphs will be constructed by plotting the ratio of peak area SCFA/peak area IS vs. concentration for each individual SCFA. By normalizing the peak area to that of the IS, the variability in the extraction step and the instrument response will be corrected (in particular, the injection volume variability and the MS response). Each point of the calibration graph shall correspond to the mean value from independent replicate injections. The limits of detection (LOD) and quantification (LOQ) of the individual analytes will be obtained by injecting successively more diluted standard solutions and will be calculated according to the International Union of Pure and Applied Chemistry 16 method [34] based on a signal-to-noise ratio (S/N) of 3 for the LOD and of 10 for the LOQ.

### 3.5. Quantification of Faecal Bile Acids

#### 3.5.1. Faecal Bulk Bile Acid Concentration

A total of 100 mg of lyophilized stool will be heated to 195°C in 1 mL of ethylene glycol KOH for 2 h, neutralized with 1 mL of saline and 0.2 mL of concentrated HCl, and then will be extracted into 6 mL of diethyl ether thrice. After the evaporation of ether, the sample residues will be dissolved in 6 mL of methanol and subjected to enzymatic analysis. The enzymatic reaction mixtures will incorporate 66.5 mmol/L Tris, 0.33 mmol/L EDTA, 0.33 mol/L hydrazine hydrate, 0.77 mmol/L NAD (N 7004, Sigma-Aldrich, St. Louis, MO, USA), 0.033 U/mL 3-hydroxysteroid dehydrogenase (Sigma-Aldrich, St. Louis, MO, USA), and either the sample or the standard (taurocholic acid; Sigma-Aldrich, St. Louis, MO, USA) dissolved in methanol. After 90 min of incubation at 37 °C, absorbance will be measured at 340 nm.

#### 3.5.2. Measurement of Primary and Secondary Bile Acids

200 mg of glass beads will be added to a suspension of ~100 mg of stool and 0.25 mL of water in a 1-dram Teflon-capped glass vial. Vortexing for 60–90 s will be performed to homogenise the suspension. Ethanol (1.8 mL) will be added, and the suspension will be heated by stirring in a heating block at 80 °C for 1.5 h. The sample will be cooled, transferred to a 2 mL Eppendorf tube, and then centrifuged at 13,500 rpm for 1–2 min. The supernatant will be removed and retained. The pellet will be resuspended in 1.8 mL of 80% aqueous ethanol, transferred to the original vial, and then heated to 80 °C for 1.5 h. The sample will be centrifuged again, and the supernatant removed and added to the first extraction supernatant. The pellet will now be resuspended in 1.8 mL of chloroform–methanol (1:1 *v*/*v*) and refluxed for 30–60 min. The sample will be centrifuged, and the supernatant will be removed and concentrated to dry on a rotary evaporator. The ethanolic supernatants will be added to the same flask, the pH will be adjusted to neutrality by adding aqueous 0.01 N HCl, and the combined extracts will be dried. The dried extract will be resuspended in 1 mL of 0.01 N aqueous HCl by sonication for 30 min. A BIO-RAD Poly-Prep chromatography column (0.8 × 4 cm) will be loaded with Lipidex 1000 as a slurry in MeOH, allowed to pack under gravity to a final volume of 1.1 mL, and will be washed with 10 mL of distilled water. The suspension will be filtered through a bed of Lipidex 1000, and the effluent will be discarded. The flask will be washed with 3 × 1 mL of 0.01 N HCl, the suspension will be passed through gel, and the bed washed with 4 mL of distilled water. Bile acids and sterols will be recovered by elution using a Lipidex gel bed with 8 mL of methanol. A BIO-RAD (Hercules, CA, USA) Poly-Prep chromatography column (0.8 × 4 cm) will be loaded with washed SP-Sephadex as a slurry in 72% aqueous MeOH to a final volume of 1.1 mL. Methanolic extract will be passed through the SP-Sephadex column, and the column will be washed with 4 mL of 72% aqueous methanol. The extract and suspension will be combined, and the pH will be brought to neutral with 0.04 N aqueous NaOH. A BIO-RAD Poly-Prep chromatography column (0.8 × 4 cm) will be loaded with Lipidex-DEAP, prepared in acetate form, as a slurry in 72% aqueous MeOH to a final volume of 1.1 mL. The combined neutralized effluent will be applied to the column, and the solution will be eluted using air gas pressure (flow rate ~25 mL/h). The flask and column were washed with 2 × 2 mL of 72% aqueous ethanol, and the sample and liquid will be combined to give a fraction of neutral compounds, including sterols. Unconjugated bile acids will be eluted using 4 mL of 0.1 M acetic acid in 72% (*v*/*v*) aqueous ethanol adjusted to pH 4.0 by the addition of concentrated ammonium hydroxide. The fraction containing bile acids will be concentrated to dry on a rotary evaporator.

The bile acids will be converted to their corresponding methyl ester derivatives by adding 0.6 mL of MeOH followed by 40 µL of 2.0 M solution of (trimethylsilyl)diazomethane in diethyl ether. The solution will be divided in half, and each half of the sample will be concentrated to dry on a rotary evaporator. Bile acids in the first half of the sample will be converted to their corresponding trimethylsilyl ether derivatives by the addition of 35 µL of 2:1 solution of N,Obis(trimethylsilyl)trifluoroacetamide and chlorotrimethylsilane, and then will be analysed by GC-MS. The identities of individual bile acids will be determined by the comparison of retention time and fragmentation pattern to known standards. Both the ratio of cholest-3-ene to deoxycholic acid in the sample and the amount of internal standard to be added shall be determined by integrating the peak areas. A known amount of internal standard, 5^®^-cholestane-3^®^-ol (5^®^-coprostanol), shall be added to the second half of the sample (0.003–0.07 mmol). Bile acids in the second half of the sample will be converted to their corresponding trimethylsilyl ether derivatives by the addition of 35 µL of 2:1 solution of N,O-bis(trimethylsilyl)trifluoroacetamide and chlorotrimethylsilane, and then will be analysed by GCMS [35,36,37]. 

### 3.6. Microbiota Analysis

#### 3.6.1. PCR Amplification and 16S rRNA Gene Sequencing

Genomic DNA will be submitted to the laboratory for library preparation and sequencing on the Illumina (San Diego, CA, USA) MiSeq platform using the MiSeq Kit v3 (2 × 300 cycles) using V3–V4 primers.

#### 3.6.2. 16S rRNA Gene Sequence Analysis

16S rRNA sequence data will be quality filtered and trimmed using TRIMMOMATIC (version 0.36) truncating reads if the quality is below 12 in a sliding window of 4 bp. USEARCH (version 10.0.240) will be used to merge and filter high-quality sequencing reads between 350 and 500 nucleotides. Unique sequences that may appear less than 8 times will be removed. Processed reads will then be concatenated and clustered into operational taxonomic units (OTU) at 97% sequence similarity using UPARSE (USEARCH version 12). Chimeras will be removed de novo in reference mode using UCHIME (version 4.2.40) together with the SILVA SSURef NR99 database (version 132), and OTU sequences will be identified taxonomically using BLASTN (BLAST + version 2.14.0) alignments against the SILVA database (version 132).

For alpha diversity measures, each sample will be subsampled 100 times to a count of 20,000 counts per sample, and the average will be taken. OTU richness and diversity indices, Simpson, Shannon, ACE, and Chao1, will be calculated in R (version 3.6.0) using the vegan package (version 2.6-8). Relative abundance analysis at the Phylum, Family, Genus, and species levels will be carried out using phyloseq package (version 1.50.0) in R 4.4. The data will be visualized using the ggplot2 (version 3.5.1) and ggpubr (version 0.6.0) packages.

For beta diversity, both the weighted UniFrac and Bray–Curtis calculations will be used. For weighted Unifrac, the data will be transformed to relative abundance. To generate a phylogenetic tree for diversity computations, zOTUs will be aligned with MAFFT (version 7.526), and the tree will be calculated with FastTree (version 2.1.11). Weighted unifrac distances will be calculated and visualized on a principal coordinate analysis (PCoA) plot. For Bray–Curtis, the data will be square root transformed, and then visualized on a non-metric multi-dimensional (NMD) scaling plot.

#### 3.6.3. Shotgun Metagenomic Analysis

The quality of the sequencing reads will be checked using FastQC (version 0.11.9). Cutadapt (version 1.8.1) will be used for the quality-filtering of raw reads to remove adapters and low-quality bases using -e 0.1, -q 20, -and a minimum-length of 30 as parameters. Bowtie2 (version 2.3.5) will be used for mapping the trimmed reads with the reference database, and GRCH38 (version GRCh38.p14) will be used to remove human host reads using “–un-conc” and “–very-sensitive-local” as parameters.

#### 3.6.4. Taxonomic Profiling

Kaiju classifier (version 1.7.0) will be used for the taxonomic classification of metagenomic reads. The database index will be created from the reference database, nr_euk, downloaded from the source. MEGAN6 will be used for calculating the normalized read counts of bacteria at the species level. To obtain sub-species information for *Bifidobacterium longum*, Kraken2 (version 2.1.3) with the default database with ‘report option’ will be used. The read counts for each taxon are the number of reads covered by the clade rooted to this taxon.

### 3.7. Statistical Analysis of Clinical, SCFA, Bile Acid and Microbiome Data

Analysis will be based on the intention-to-treat principle. The continuous data will be summarized using median, interquartile range, and range. The categorical data will be summarized using frequency distributions. Univariate comparisons for the continuous data will be made using the Mann–Whitney test. For the categorical data, the Chi-square or Fisher exact test will be used.

The SCFA data will be analysed using a linear mixed model effect (LME) test to assess the differences between the groups over time. In this analysis, the subjects will be modelled as a random factor, and time and treatment are fixed factors. All the tests will be two-sided, and a *p*-value < 0.05 will be considered significant. The data on clinical outcomes will be analysed using SPSS version 22.0 statistical software (Armonk, NY, USA: IBM Corp.) and SCFA in R (version 3.5.1).

**Pre-planned subgroups:** (1) Infants who are born through caesarean section due to increased risk of gut dysbiosis. (2) Infants born to mothers with diet-controlled vs. insulin-dependent GDM. (3) Infants born to mothers with GDM and a normal vs. high BMI.

## 4. Discussion

To our knowledge, the PRIMD trial is the first adequately powered, placebo-controlled RCT evaluating the effect of probiotic supplementation on dysbiosis in infants of mothers with GDM and the risk of probiotic sepsis.

Given the adverse effects of maternal GDM on infants’ gut microbiota and neurodevelopment, it is important to assess this outcome in our trial [38,39,40,41]. The dynamic maturation of gut microbiota during infancy is affected by various factors, including maternal health (e.g., GDM), the delivery mode, the feeding pattern, gestational age, and exposure to medications [42,43]. The disruption of initial microbial colonization has been associated with risk of adverse neurodevelopment, allergies, obesity, and auto-immune disorders in later life [44]. Gut microbiota and their metabolites regulate neurodevelopment and cognitive functioning via the bi-directional communication system known as the GMB axis [45]. The GMB axis influences brain development and function via the hypothalamo–pituitary axis (HPA); the vagus nerve; immune signalling; the production of neurotransmitters; and the production of metabolites, such as SCFAs (butyrate, propionate, and acetate), bile acids, and tryptophan derivatives. SCFAs maintain and modulate both gut epithelial and blood–brain barrier (BBB) integrity [46,47,48]. They directly affect the brain, binding to local receptors and induce epigenetic modifications, thus modulating neuro-inflammation. Additionally, SCFAs’ enhance regulatory T cell function and activate the sympathetic nervous system and the vagus nerve. Indole, a tryptophan derivative, reduces neuro-inflammation and enhances gut epithelial and BBB integrity [49]. Bile acids influence the brain and HPA axis through specific local receptor modulation [50,51]. Gut microbiota can produce serotonin, dopamine, nor-epinephrine, histamine, and gamma-amino butyric acid (GABA) directly or indirectly via entero-endocrine cell stimulation in the gut epithelium. Bifidobacteria are known to produce GABA [52]. These neurotransmitters exert local effects through vagal stimulation and can directly affect the brain by traversing the BBB [53].

The association of microbiota composition and neurodevelopment has been reported in term infants not supplemented with probiotics. A recent study reported an association between increased levels of Bifidobacteria and Eggerthella and decreased levels of Streptococcus, and Hungatella, linked to a successful performance on the Point-and-Gaze attention test in 44 term infants at 4–6 months age [54]. Bonham et al. reported the influence of Klebsiella, *E. coli*, and Bifidobacteria in the gut in the first year of life and the prediction of cognitive function and brain growth via MRI [55]. Zhou et al. showed significantly higher neurodevelopmental scores at 6 months following vaginal microbiota transfer in 68 term infants born via caesarean section [56]. Furthermore, Bifidobacterial dominance in the first 1000 days of life has shown positive influences on cognition, adaptive behaviour, and communication in preterm-born children [57]. Given this evidence, probiotic supplementation may provide a pathway to modulate the GMB axis and improve neurocognition in infants of mothers with GDM.

GDM during pregnancy adversely impacts infant weight gain and increases the risk of obesity in the long term [54,55,56]. Obesity is a multifactorial disorder influenced by genetic, environmental, lifestyle factors, and possibly the microbiome [58,59,60]. Furthermore, the Developmental Origins of Health and Disease (DOHAD) hypothesis highlights the role of an unfavourable ‘in utero’ environment on long-term health, particularly metabolic syndrome and cardiovascular disease later in life [61,62,63,64]. The modulation of gut microbiota has been shown to be effective treatment for obesity and seems to be a promising strategy [65,66].

If proven effective in improving dysbiosis, future studies should investigate the optimal timing, dosage, strain, and duration of probiotic supplementation during the perinatal period in infants of mothers with GDM. Additionally, data linkage studies assessing the long-term effects of early microbiota modulation would provide evidence of the effectiveness of probiotic supplementation beyond merely improving dysbiosis in such infants.

## 5. Conclusions

We believe our innovative trial assessing the effects of probiotic supplementation on dysbiosis in the infants of mothers with GDM will provide robust data on gut microbiota, faecal short-chain fatty acids, long-term growth, and neurodevelopmental outcomes to guide adequately powered robust RCTs in this field.

## Data Availability

The original contributions presented in this study are included in the article. Further inquiries can be directed to the corresponding authors.

## References

[1] Zhou T., Du S., Sun D., Li X., Heianza Y., Hu G., Sun L., Pei X., Shang X., Qi L. (2022). Prevalence and Trends in Gestational Diabetes Mellitus Among Women in the United States, 2006–2017: A Population-Based Study. Front. Endocrinol..

[2] Wang H., Li N., Chivese T., Werfalli M., Sun H., Yuen L., Hoegfeldt C.A., Elise Powe C., Immanuel J., Karuranga S. (2022). IDF Diabetes Atlas: Estimation of Global and Regional Gestational Diabetes Mellitus Prevalence for 2021 by International Association of Diabetes in Pregnancy Study Group’s Criteria. Diabetes Res. Clin. Pract..

[3] McIntyre H.D., Sweeting A. (2022). Gestational diabetes in Australia: Navigating a tsunami. Lancet Diabetes Endocrinol..

[4] Dias S., Pheiffer C., Adam S. (2023). The Maternal Microbiome and Gestational Diabetes Mellitus: Cause and Effect. Microorganisms.

[5] Fan Y., Pedersen O. (2021). Gut microbiota in human metabolic health and disease. Nat. Rev. Microbiol..

[6] Ferretti P., Pasolli E., Tett A., Asnicar F., Gorfer V., Fedi S., Armanini F., Truong D.T., Manara S., Zolfo M. (2018). Mother-to-Infant Microbial Transmission from Different Body Sites Shapes the Developing Infant Gut Microbiome. Cell Host Microbe.

[7] Bäckhed F., Roswall J., Peng Y., Feng Q., Jia H., Kovatcheva-Datchary P., Li Y., Xia Y., Xie H., Zhong H. (2015). Dynamics and Stabilization of the Human Gut Microbiome during the First Year of Life. Cell Host Microbe.

[8] Soderborg T.K., Carpenter C.M., Janssen R.C., Weir T.L., Robertson C.E., Ir D., Young B.E., Krebs N.F., Hernandez T.L., Barbour L.A. (2020). Gestational Diabetes Is Uniquely Associated with Altered Early Seeding of the Infant Gut Microbiota. Front. Endocrinol..

[9] Wang J., Zheng J., Shi W., Du N., Xu X., Zhang Y., Ji P., Zhang F., Jia Z., Wang Y. (2018). Dysbiosis of maternal and neonatal microbiota associated with gestational diabetes mellitus. Gut.

[10] Chen T., Qin Y., Chen M., Zhang Y., Wang X., Dong T., Chen G., Sun X., Lu T., White R.A. (2021). Gestational diabetes mellitus is associated with the neonatal gut microbiota and metabolome. BMC Med..

[11] Notarbartolo V., Carta M., Accomando S., Giuffrè M. (2023). The First 1000 Days of Life: How Changes in the Microbiota Can Influence Food Allergy Onset in Children. Nutrients.

[12] Sarkar A., Yoo J.Y., Valeria Ozorio Dutra S., Morgan K.H., Groer M. (2021). The Association between Early-Life Gut Microbiota and Long-Term Health and Diseases. J. Clin. Med..

[13] Rowland J., Wilson C.A. (2021). The association between gestational diabetes and ASD and ADHD: A systematic review and meta-analysis. Sci. Rep..

[14] Kang D.W., Park J.G., Ilhan Z.E., Wallstrom G., Labaer J., Adams J.B., Krajmalnik-Brown R. (2013). Reduced incidence of Prevotella and other fermenters in intestinal microflora of autistic children. PLoS ONE.

[15] Su M., Nie Y., Shao R., Duan S., Jiang Y., Wang M., Xing Z., Sun Q., Liu X., Xu W. (2018). Diversified gut microbiota in newborns of mothers with gestational diabetes mellitus. PLoS ONE.

[16] Davidson S.J., Barrett H.L., Price S.A., Callaway L.K., Dekker Nitert M. (2021). Probiotics for preventing gestational diabetes. Cochrane Database Syst. Rev..

[17] Valiati N., Puel E.M., Stefani C.M., Lataro R.M. (2024). Does probiotic ingestion reduce the risk of preeclampsia? A systematic review. Appl. Physiol. Nutr. Metab..

[18] Saturio S., Nogacka A.M., Alvarado-Jasso G.M., Salazar N., de Los Reyes-Gavilán C.G., Gueimonde M., Arboleya S. (2021). Role of Bifidobacteria on Infant Health. Microorganisms.

[19] Athalye-Jape G., Esvaran M., Patole S., Simmer K., Nathan E., Doherty D., Keil A., Rao S., Chen L., Chandrasekaran L. (2022). Effect of single versus multistrain probiotic in extremely preterm infants: A randomised trial. BMJ Open Gastroenterol..

[20] Rao S., Esvaran M., Chen L., Keil A.D., Gollow I., Simmer K., Wemheuer B., Conway P., Patole S. (2022). Probiotic supplementation in neonates with congenital gastrointestinal surgical conditions: A pilot randomised controlled trial. Pediatr. Res..

[21] Link V.M., Subramanian P., Cheung F., Han K.L., Stacy A., Chi L., Sellers B.A., Koroleva G., Courville A.B., Mistry S. (2024). Differential peripheral immune signatures elicited by vegan versus ketogenic diets in humans. Nat. Med..

[22] Wang X.A., Li J.P., Lee M.S., Yang S.F., Chang Y.S., Chen L., Li C.W., Chao Y.H. (2024). A common trajectory of gut microbiome development during the first month in healthy neonates with limited inter-individual environmental variations. Sci. Rep..

[23] John D., Michael D., Dabcheva M., Hulme E., Illanes J., Webberley T., Wang D., Plummer S. (2024). A double-blind, randomized, placebo-controlled study assessing the impact of probiotic supplementation on antibiotic induced changes in the gut microbiome. Front. Microbiomes.

[24] Homann C.M., Rossel C.A.J., Dizzell S., Bervoets L., Simioni J., Li J., Gunn E., Surette M.G., de Souza R.J., Mommers M. (2021). Infants’ First Solid Foods: Impact on Gut Microbiota Development in Two Intercontinental Cohorts. Nutrients.

[25] Panchal H., Athalye-Jape G., Rao S., Patole S. (2023). Growth and neuro-developmental outcomes of probiotic supplemented preterm infants-a systematic review and meta-analysis. Eur. J. Clin. Nutr..

[26] Catania J., Pandit N.G., Ehrlich J.M., Zaman M., Stone E., Franceschi C., Smith A., Tanner-Smith E., Zackular J.P., Bhutta Z.A. (2021). Probiotic Supplementation for Promotion of Growth in Children: A Systematic Review and Meta-Analysis. Nutrients.

[27] Indrio F., Gutierrez Castrellon P., Vandenplas Y., Cagri Dinleyici E., Francavilla R., Mantovani M.P., Grillo A., Beghetti I., Corvaglia L., Aceti A. (2022). Health Effects of Infant Formula Supplemented with Probiotics or Synbiotics in Infants and Toddlers: Systematic Review with Network Meta-Analysis. Nutrients.

[28] Lin F.-L., Chen C.-M., Sun C.-K., Cheng Y.-S., Tzang R.-F., Chiu H.-J., Wang M.-Y., Cheng Y.-C., Hung K.-C. (2023). Effects of probiotics on neurocognitive outcomes in infants and young children: A meta-analysis. Front. Public. Health.

[29] Zhang D., Lan Y., Zhang J., Cao M., Yang X., Wang X. (2024). Effects of early-life gut microbiota on the neurodevelopmental outcomes of preterm infants: A multi-center, longitudinal observational study in China. Eur. J. Pediatr..

[30] Acuña I., Cerdó T., Ruiz A., Torres-Espínola F.J., López-Moreno A., Aguilera M., Suárez A., Campoy C. (2021). Infant Gut Microbiota Associated with Fine Motor Skills. Nutrients.

[31] Kulkarni T., Majarikar S., Deshmukh M., Ananthan A., Balasubramanian H., Keil A., Patole S. (2022). Probiotic sepsis in preterm neonates-a systematic review. Eur. J. Pediatr..

[32] Blackford K., Crawford G., Burns S. (2024). Implications of the latest release of the National Statement on Ethical Conduct in Human Research on health promotion practice in Australia. Health Promot. J. Aust..

[33] Moher D., Schulz K.F., Altman D.G., CONSORT Group (2003). The CONSORT statement: Revised recommendations for improving the quality of reports of parallel-group randomised trials. Clin. Oral Investig..

[34] Allegrini F., Olivieri A.C. (2014). IUPAC-consistent approach to the limit of detection in partial least-squares calibration. Anal. Chem..

[35] Porter J.L., Fordtran J.S., Santa Ana C.A., Emmett M., Hagey L.R., MacDonald E.A., Hofmann A.F. (2003). Accurate enzymatic measurement of fecal bile acids in patients with malabsorption. J. Lab Clin. Med..

[36] Setchell K.D., Lawson A.M., Tanida N., Sjovall J. (1983). General methods for the analysis of metabolic profiles of bile acids and related compounds in feces. J. Lipid Res..

[37] David L.A., Maurice C.F., Carmody R.N., Gootenberg D.B., Button J.E., Wolfe B.E., Ling A.V., Devlin A.S., Varma Y., Fischbach M.A. (2014). Diet rapidly and reproducibly alters the human gut microbiome. Nature.

[38] Torres Y., Celis C., Acurio J., Escudero C. (2023). Language Impairment in Children of Mothers with Gestational Diabetes, Preeclampsia, and Preterm Delivery: Current Hypothesis and Potential Underlying Mechanisms: Language Impartment and Pregnancy Complications. Adv. Exp. Med. Biol..

[39] Su C.H., Liu T.Y., Chen I.T., Ou-Yang M.C., Huang L.T., Tsai C.C., Chen C.C. (2021). Correlations between serum BDNF levels and neurodevelopmental outcomes in infants of mothers with gestational diabetes. Pediatr. Neonatol..

[40] Saros L., Lind A., Setänen S., Tertti K., Koivuniemi E., Ahtola A., Haataja L., Shivappa N., Hébert J.R., Vahlberg T. (2023). Maternal obesity, gestational diabetes mellitus, and diet in association with neurodevelopment of 2-year-old children. Pediatr. Res..

[41] Sun G., Liu Y., Zhang R., Peng C., Geng Y., Zhou F., Hou X., Liu L. (2022). Emotional Prosodies Processing and Its Relationship With Neurodevelopment Outcome at 24 Months in Infants of Diabetic Mothers. Front. Pediatr..

[42] Bresesti I., Salvatore S., Valetti G., Baj A., Giaroni C., Agosti M. (2022). The Microbiota-Gut Axis in Premature Infants: Physio-Pathological Implications. Cells.

[43] Laue H.E., Coker M.O., Madan J.C. (2022). The Developing Microbiome From Birth to 3 Years: The Gut-Brain Axis and Neurodevelopmental Outcomes. Front. Pediatr..

[44] Akagawa S., Kaneko K. (2022). Gut microbiota and allergic diseases in children. Allergol. Int..

[45] Cryan J.F., O’Riordan K.J., Cowan C.S.M., Sandhu K.V., Bastiaanssen T.F.S., Boehme M., Codagnone M.G., Cussotto S., Fulling C., Golubeva A.V. (2019). The Microbiota-Gut-Brain Axis. Physiol. Rev..

[46] Aburto M.R., Cryan J.F. (2024). Gastrointestinal and brain barriers: Unlocking gates of communication across the microbiota-gut-brain axis. Nat. Rev. Gastroenterol. Hepatol..

[47] Gars A., Ronczkowski N.M., Chassaing B., Castillo-Ruiz A., Forger N.G. (2021). First Encounters: Effects of the Microbiota on Neonatal Brain Development. Front. Cell Neurosci..

[48] Silva Y.P., Bernardi A., Frozza R.L. (2020). The Role of Short-Chain Fatty Acids From Gut Microbiota in Gut-Brain Communication. Front. Endocrinol..

[49] Zhou Q., Niño D.F., Yamaguchi Y., Wang S., Fulton W.B., Jia H., Lu P., Prindle T., Pamies D., Morris M. (2021). Necrotizing enterocolitis induces T lymphocyte-mediated injury in the developing mammalian brain. Sci. Transl. Med..

[50] Li L., Yang J., Liu T., Shi Y. (2023). Role of the gut-microbiota-metabolite-brain axis in the pathogenesis of preterm brain injury. Biomed. Pharmacother..

[51] Naspolini N.F., Schüroff P.A., Figueiredo M.J., Sbardellotto G.E., Ferreira F.R., Fatori D., Polanczyk G.V., Campos A.C., Taddei C.R. (2024). The Gut Microbiome in the First One Thousand Days of Neurodevelopment: A Systematic Review from the Microbiome Perspective. Microorganisms.

[52] Duranti S., Ruiz L., Lugli G.A., Tames H., Milani C., Mancabelli L., Mancino W., Longhi G., Carnevali L., Sgoifo A. (2020). Bifidobacterium adolescentis as a key member of the human gut microbiota in the production of GABA. Sci. Rep..

[53] Frerichs N.M., de Meij T.G.J., Niemarkt H.J. (2024). Microbiome and its impact on fetal and neonatal brain development: Current opinion in pediatrics. Curr. Opin. Clin. Nutr. Metab. Care.

[54] Hunter S., Flaten E., Petersen C., Gervain J., Werker J.F., Trainor L.J., Finlay B.B. (2023). Babies, bugs and brains: How the early microbiome associates with infant brain and behavior development. PLoS ONE.

[55] Bonham K.S., Fahur Bottino G., McCann S.H., Beauchemin J., Weisse E., Barry F., Cano Lorente R., Huttenhower C., Bruchhage M., D’Sa V. (2023). Gut-resident microorganisms and their genes are associated with cognition and neuroanatomy in children. Sci. Adv..

[56] Zhou L., Qiu W., Wang J., Zhao A., Zhou C., Sun T., Xiong Z., Cao P., Shen W., Chen J. (2023). Effects of vaginal microbiota transfer on the neurodevelopment and microbiome of cesarean-born infants: A blinded randomized controlled trial. Cell Host Microbe.

[57] Sadeghpour Heravi F., Hu H. (2023). Bifidobacterium: Host–microbiome interaction and mechanism of action in preventing common gut-microbiota-associated complications in preterm infants: A narrative review. Nutrients.

[58] Franzago M., Borrelli P., Di Nicola M., Cavallo P., D’Adamo E., Di Tizio L., Gazzolo D., Stuppia L., Vitacolonna E. (2024). From Mother to Child: Epigenetic Signatures of Hyperglycemia and Obesity during Pregnancy. Nutrients.

[59] Wang S.S., Yue Z.H., Han N., Lyu J.L., Ji Y.L., Wang H., Liu J., Wang H.J. (2024). Association of maternal pre-pregnancy BMI, gestational weight gain, and gestational diabetes mellitus with BMI trajectory in early childhood: A prospective cohort study. Zhonghua Liu Xing Bing Xue Za Zhi.

[60] Malik N., Ahmad A., Ashraf H. (2024). Metabolic Profile of Offspring of Mothers with Gestational Diabetes Mellitus. Indian J. Endocrinol. Metab..

[61] Zhang S., Dang Y. (2022). Roles of gut microbiota and metabolites in overweight and obesity of children. Front. Endocrinol..

[62] Kvit K.B., Kharchenko N.V. (2017). Gut microbiota changes as a risk factor for obesity. Wiad. Lek..

[63] Vallès Y., Arshad M., Abdalbaqi M., Inman C.K., Ahmad A., Drou N., Gunsalus K.C., Ali R., Tahlak M., Abdulle A. (2024). The infants’ gut microbiome: Setting the stage for the early onset of obesity. Front. Microbiol..

[64] Charles M.A., Delpierre C., Bréant B. (2016). Developmental origin of health and adult diseases (DOHaD): Evolution of a concept over three decades. Med. Sci..

[65] Ridaura V.K., Faith J.J., Rey F.E., Cheng J., Duncan A.E., Kau A.L., Griffin N.W., Lombard V., Henrissat B., Bain J.R. (2013). Gut microbiota from twins discordant for obesity modulate metabolism in mice. Science.

[66] Le Chatelier E., Nielsen T., Qin J., Prifti E., Hildebrand F., Falony G., Almeida M., Arumugam M., Batto J.M., Kennedy S. (2013). Richness of human gut microbiome correlates with metabolic markers. Nature.

